# Metallic allergy requiring removal of iliac stent: report of a case

**DOI:** 10.1186/s40792-020-00843-8

**Published:** 2020-04-26

**Authors:** Atsushi Guntani, Eisuke Kawakubo, Ryosuke Yoshiga, Shinsuke Mii

**Affiliations:** Department of Vascular Surgery, Saiseikai Yahata General Hospital, 5-9-27 Haruno-machi, Yahatahigashi-ku, Kitakyushu, 805-8527 Japan

**Keywords:** Contact dermatitis, Nickel allergy, Metallic stent, Iliac stent

## Abstract

**Background:**

The use of metallic stents, which are predominantly composed of nickel, in the treatment of patients with nickel allergy has not been well studied.

**Case presentation:**

A patient who suffered from contact dermatitis due to implantation of an iliac stent was successfully treated by removing the stent that caused nickel allergy. The patient has exhibited no symptoms of claudication or severe pruritic rash in the 2-year follow-up period after iliac stent removal.

**Conclusions:**

We herein report a case of nickel allergy in which a metallic iliac stent that was predominantly composed of nickel was removed. The patient showed a marked recovery from her contact dermatitis. In cases of suspected metallic allergy, it is necessary to consider revascularization without using a metal device.

## Background

Endovascular treatment has already become an indispensable procedure in peripheral artery disease. Especially in the treatment of iliac artery lesions, metallic stents are the first choice. Nickel is a common allergen in patients with contact dermatitis and metallic stents are predominantly composed of this metal [1–3]. Iliac stenting for patients with nickel allergy has not been well studied in the literature. We successfully treated a patient with nickel allergy by removing a metallic stent from the patient’s iliac artery.

## Case presentation

**Fig. 1:**
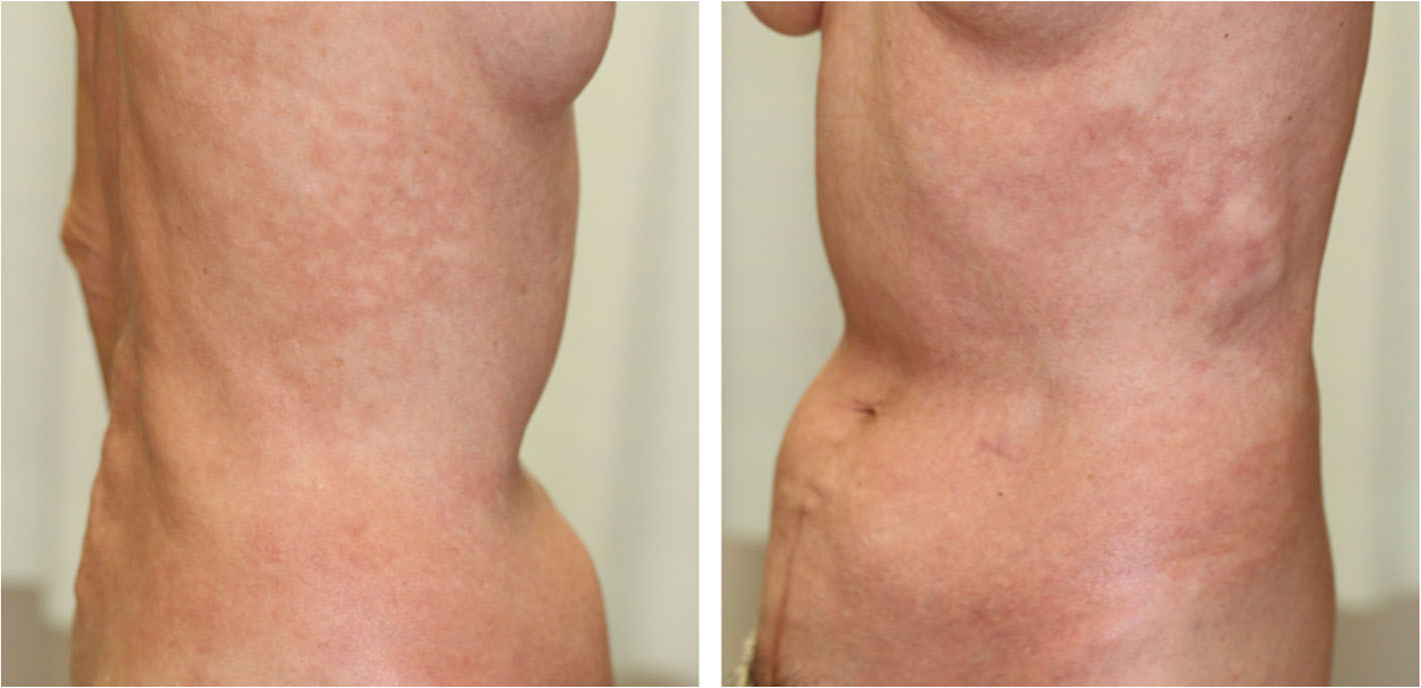
A picture on admission showing rash around the abdomen

**Fig. 2:**
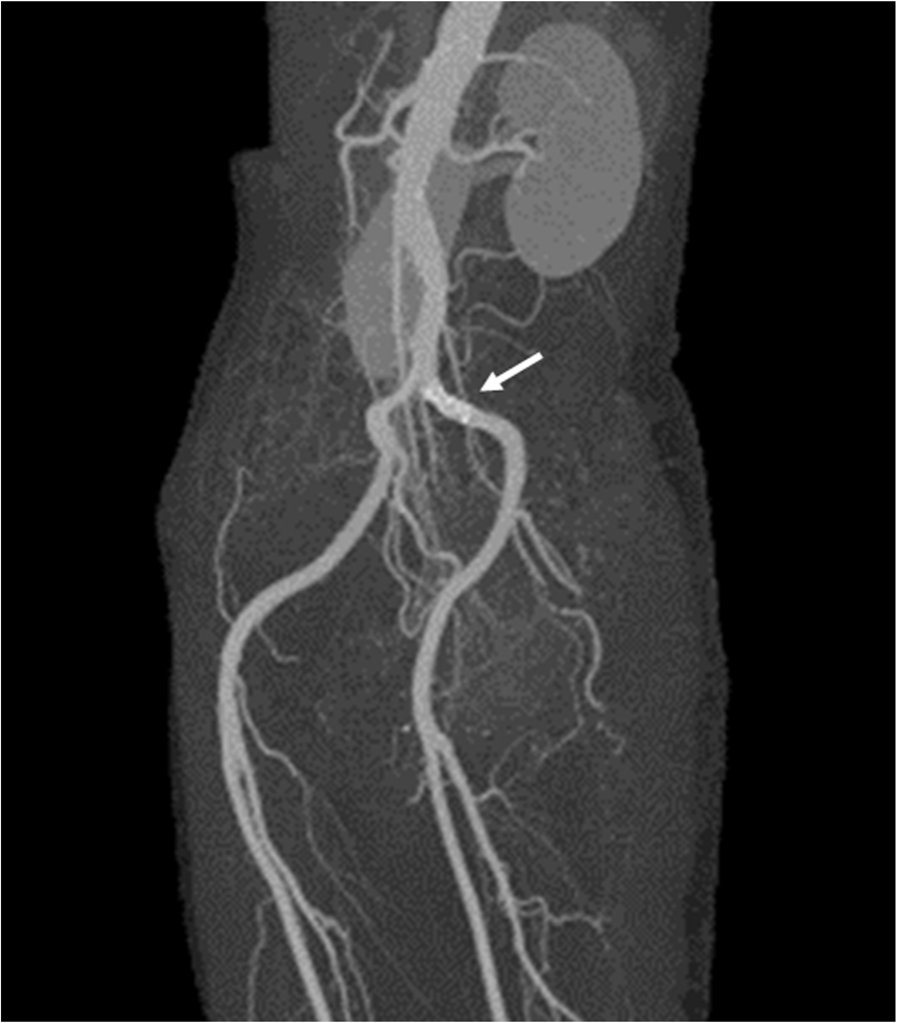
On contrast-enhanced computed tomography the stent (arrow) implanted in the left common iliac artery showed good patency

A 70-year-old female patient was admitted to our hospital for generalized pruritus and rash (Fig. 1). She had suffered from left calf claudication for several years. Endovascular treatment using a balloon-expandable metallic stent (Express^TM^ LD; Boston Scientific, San Diego, CA, USA) had been performed to treat left common iliac artery stenosis 4 years prior to her hospitalization (Fig. 2). Shortly after stenting, a band-like erythema was observed around the navel, which gradually worsened into a generalized rash, burning, and abdominal pain that persisted for 10 months. Finally, she suffered from depression. She had undergone patch tests at several medical institutions, including university hospitals, and was diagnosed with contact dermatitis due to the nickel contained in the stent. Since the administration of steroids did not improve the symptoms, it was determined that the removal of the allergen was appropriate.

We performed an operation to remove the iliac artery stent. On entering the abdominal cavity from an upper to lower midline skin incision, calcification was observed in the bilateral common iliac arteries; therefore, the terminal aorta, bilateral common iliac arteries, left internal, and external iliac artery were all controlled by clamping. The left common iliac artery was incised longitudinally and the stent (measuring 25 mm in length), which was covered with the intima, was removed. Reconstruction was performed with simple closure of the adventitia. The findings of two-year follow-up computed tomography showed no aneurysm or stenosis after revascularization.

**Fig. 3:**
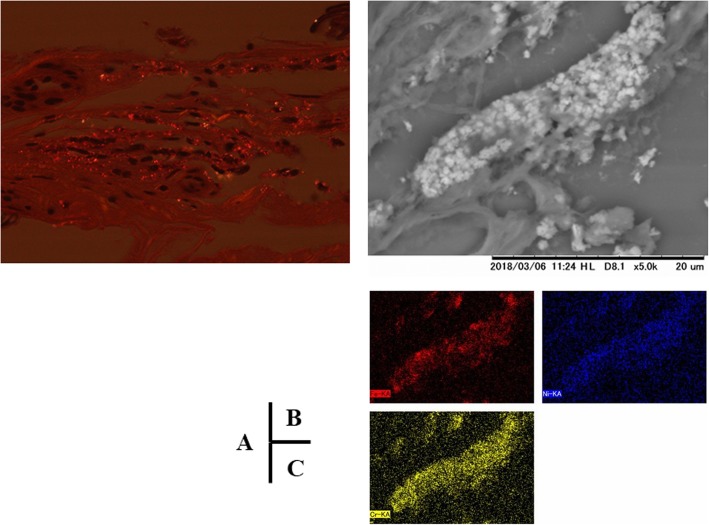
Infiltration of inflammatory cells and eosinophils was not found; however, concentrations of histocytes that phagocytosed black fine granules with heavy refraction were observed in some places (**a**). Energy dispersed X-ray spectroscopy revealed that the deposits consisted of nickel (Ni), chromium (Cr) and iron/ferrum (Fe) (**b**, **c**)

The pathological examination of the removed metallic stent demonstrated fibrointimal proliferation with calcification, without infiltration of inflammatory cells or eosinophils. Concentrations of histocytes that phagocytosed black fine granules with heavy refraction were observed in some places. Energy dispersed X-ray spectroscopy revealed that the deposits consisted of nickel (Ni), chromium (Cr), and iron/ferrum (Fe) (Fig. 3).

Postoperatively, her dermatitis significantly improved, and her depression disappeared immediately. Follow-up computed tomography showed no recurrence of stenosis of the left iliac artery or any other inflammation. The patient remained free of symptoms of claudication and severe pruritic rash in the 2-year follow-up period after iliac stent removal.

## Discussion

Endovascular treatment of the iliac artery with metallic stent usually shows favorable short-term and long-term clinical outcomes. The incidence and prevalence of allergic reaction to implanted metals are unknown. According to a recent case series, it is estimated that up to 5% of patients had metal-related cutaneous complications after implantation, including dental implants, orthopedic joints/static implants and pacemakers/defibrillators, and nickel is reported to be the most common allergen with 18.5% of patients that are patch tested having a positive reaction [1, 4, 5].

Regarding the mechanism of allergic contact dermatitis, it has been reported that after the implantation of a stent that contains nickel, the metal is steadily released into the systemic circulation, promoting the recruitment of inflammatory cells from the bloodstream. In addition, local exposure to a stent containing nickel causes a type IV delayed hypersensitivity reaction mediated by allergen-specific T lymphocytes, which can trigger excessive immunologic reactions [1, 6]. Although our patient was aware of skin irritation relatively soon after the implantation of the metallic stent, the diagnosis of her metal allergy was time-consuming due to the rare condition, and tests to rule out the presence of either rheumatic diseases or neurological diseases were conducted at several medical institutions. In addition, since it is not common to remove an implanted stent, the patient developed depressive symptoms due to the inability to remove the stent easily have been added, and the patient’s condition has deteriorated.

As revealed in a later interview, she had been previously aware of allergic hypersensitivity to metal jewelry before the implantation of the iliac stent. A patch test was positive for nickel, and symptoms resolved after iliac stent removal, we have determined that the cause of her dermatitis was nickel allergy. In addition, the detection of nickel in the explanted specimen supports our definitive diagnosis of nickel allergy.

Regarding the method of reconstruction after stent removal, in some case reports, bypass surgery using an autologous vein was performed; however, in our case, direct suturing was possible since the stent could be removed while leaving sufficient vascular wall, and the suturing distance was short [7, 8]. In fact, the findings of two-year follow-up computed tomography showed no aneurysm or stenosis after revascularization, direct suturing seemed to be a good reconstruction method without leaving any artifacts for this case.

We realized that prior to device implantation, metal allergies should be discussed routinely, and metal skin patch tests should be considered in cases in which metal hypersensitivity is suspected, such as our case. When a patient with symptoms of lower limb ischemia is suspected of having a metal allergy, revascularization without metal devices should be considered.

## Conclusion

We reported a rare case of contact dermatitis caused by nickel allergy due to the implantation of a metallic stent for iliac stenosis that was successfully treated by removing the allergenic stent.

## Data Availability

All datasets supporting the conclusions of this article are included in this published article.
